# Factors Affecting the Analytical Performance of Magnetic Molecularly Imprinted Polymers

**DOI:** 10.3390/polym14153008

**Published:** 2022-07-25

**Authors:** Nur Masyithah Zamruddin, Herman Herman, Laode Rijai, Aliya Nur Hasanah

**Affiliations:** 1 Department of Pharmaceutical Analysis and Medicinal Chemistry, Faculty of Pharmacy, Padjadjaran University, Jl. Raya Bandung Sumedang KM 21, Sumedang 45363, Indonesia; nur21050@mail.unpad.ac.id; 2 Department of Pharmaceutical Chemistry, Faculty of Pharmacy, Mulawarman University, Gunung Kelua 75119, Indonesia; herman@farmasi.unmul.ac.id (H.H.); rijai@farmasi.unmul.ac.id (L.R.); 3 Drug Development Study Centre, Faculty of Pharmacy, Padjadjaran University, Jl. Raya Bandung Sumedang KM 21, Sumedang 45363, Indonesia

**Keywords:** magnetic molecularly imprinted polymer (MMIP), factors affecting MMIP, components of MMIP, magnetic separation technology

## Abstract

During the last few years, separation techniques using molecular imprinting polymers (MIPs) have been developed, making certain improvements using magnetic properties. Compared to MIP, Magnetic molecularly imprinted polymers (MMIPs) have high selectivity in sample pre-treatment and allow for fast and easy isolation of the target analyte. Its magnetic properties and good extraction performance depend on the MMIP synthesis step, which consists of 4 steps, namely magnetite manufacture, magnetic coating using modified components, polymerization and template desorption. This review discusses the factors that will affect the performance of MMIP as a selective sorbent at each stage. MMIP, using Fe_3_O_4_ as a magnetite core, showed strong superparamagnetism; it was prepared using the co-precipitation method using FeCl_3_·6H_2_O and FeCl_2_·H_2_O to obtain high magnetic properties, using NH_4_OH solution added for higher crystallinity. In magnetite synthesis, the use of a higher temperature and reaction time will result in a larger nanoparticle size and high magnetization saturation, while a higher pH value will result in a smaller particle size. In the modification step, the use of high amounts of oleic acid results in smaller nanoparticles; furthermore, determining the correct molar ratio between FeCl_3_ and the shielding agent will also result in smaller particles. The next factor is that the proper ratio of functional monomer, cross-linker and solvent will improve printing efficiency. Thus, it will produce MMIP with high selectivity in sample pre-treatment.

## 1. Introduction

Imprinting technology provides the basis for molecular recognition to design coordinated, specific, selectively identified sites within synthetic polymer systems. Molecular imprinted technology (MIT) is seen as an effective and efficient approach to achieve molecular recognition functions [1,2] and is a method for producing synthetic materials such as artificial receptors (molecularly imprinted polymers, MIPs), which are obtained by generating a memory of the printed molecule in the form of the size, shape and functional group of the imprint molecule [3,4]. The most widely used methods in the manufacture of MIPs are free radical polymerisation (FRP) methods, namely bulk polymerisation, suspension polymerisation, emulsion or precipitation polymerisation, and the sol-gel method [5]. However, MIPs prepared by the common FRP method have several disadvantages, such as slow mass transfer, irregular shape, imperfect removal of the template molecule, poor site accessibility and/or heterogeneous distribution of the binding sites [6]. Efforts were made to overcome these problems by implanting a magnet during the manufacture of the MIP and performing magnetic separation [7].

Magnetic separation technology, in which polymers are prepared using MIP fabrication on the surface of a magnetic substrate, has been widely used in recent years for separation and extraction applications [8,9,10,11], such as in the field of drug analysis in biological fluids [12,13,14,15,16,17], analysis of compounds in the environment [9,10,11,18,19,20,21,22], analysis of compounds in food [8,23,24,25] and analysis of compounds in plants and other naturally occurring products [26,27,28,29]. Magnetic molecular imprinted solid phase extraction (MMI-SPE) is a new solid phase extraction (SPE) procedure based on the use of magnetic sorbents [8,16]. Magnetic molecularly imprinted polymers (MMIPs) have the advantages of fast and effective binding to the target analyte, spherical shaped sorbents that exhibit magnetic properties, highly selective binding to target imprinted molecules and analogues, easy isolation from samples using magnets via external filtering or centrifugation steps, shorter pre-treatment times, reversible and controlled flocculation, and easy separation of polymers from the sample matrix using external magnets [9,30,31].

Several MMIP technologies have been successfully applied to several compounds: MMIPs based on surface MIT have been used for the extraction of norfloxacin in water samples, with an absorption capacity of 82.7% for non-imprinted polymer (NIP) and 91.1% for MMIP [3]; a MMIP has also been synthesised on the surface of chitosan-Fe_3_O_4_ by precipitation polymerisation for the extraction of tricyclazole from rice and water samples, with a binding capacity of 45,454.55 g/g compared to 26,315.79 g/g for the NIP [31]; and a MMIP has been synthesised and modified using oleic acid as a surfactant for the extraction of chloramphenicol from honey samples, showed the value of the dissociation constant of 329.9 lmol/L and the maximum binding capacity of 17.1 lmol/g, compared to magnetic non-imprinted polymer (MNIP) with values of dissociation constant 217.2 mol/L and maximum binding capacity 8.8 mol/g [8].

MMIP preparation begins with the preparation of a magnetic core, commonly called magnetite, using a co-precipitation technique between ferric chloride (FeCl_2_·H_2_O) or ferrous sulphate (FeSO_4_·7H_2_O) and iron(III) chloride hexahydrate (FeCl_3_·6H_2_O), which can be achieved under basic conditions of 80–100 °C [14]. After the magnetite is formed, its surface is modified, either by silanisation or by adding a surfactant such as ethylene glycol or oleic acid, enhancing the amphoteric properties of the magnetite surface and improving its interaction with polar solutions. The modified magnetite is then polymerized using a template, functional polymer or cross-linker. The final step in the manufacture of MMIP is the desorption of the template molecules from the polymer. Combining magnetic separation with molecular imprinting would be ideal, providing a powerful analytical tool for use in separation [32].

The type of magnetic particle used affects the yield of the magnetic core particles created. [13], and temperature, reaction time [33], initial concentration of ferric chloride (FeCl_3_) [34], pH value and surfactants [35] will also influence the magnetic core that results. After synthesis of the magnetic core, the core-shell is usually modified, and the components used will influence the size of the final particles [33]; oleic acid is used more widely as it results in smaller nanoparticles (NPs) [36]. At the polymerisation stage, the selection of the polymerisation component and the ratio have to considered [17,37,38]. The amount of MMIP, extraction time, washing and eluent conditions affect the results of template extraction which is the final stage in making MMIP with good performance [8].

There have been MMIP review articles discussing the synthesis and application of MIPs, recent configurations and progressive use of magnetically imprinted polymers for drug analysis [13], and the design and characterization, toxicity and biocompatibility of magnetic nanoparticles (MNPs) [39]. There have also been reviews on magnetic molecularly imprinted electrochemical sensors [40], magnetic solids in analytical chemistry [41] and updates on the use of MMIPs in the separation of active compounds [27]. However, there are no review articles that specifically address the factors affecting the performance of MMIPs in an effort to produce the desired shape and performance of MMIP.Hence, this review will discuss the factors relating to the production of MMIPs with good analytical performance.

## 2. Synthesis of MMIP

Magnetic nanoparticles expressing a unique surface effect with super-magnetism properties, easy modification by functional groups, non-toxic properties, and availability in abundant quantities are able to assist in synthesizing on a large scale an efficient recycling process for efficient water purification processes. Magnetic properties can be obtained by VSM studies analyzing hysteresis loops (MH) which shows values for saturation magnetization (Ms), remanent magnetization (Mr), and coervicity (HC). Iron oxide nanoparticles have an Ms value of more than 1 emu/g, indicating that the material has good magnetic separation ability. However, magnetic nanoparticles have a strong tendency to oxidize on contact with air and exhibit Fe_3_O_4_ leaching, limiting their applicability in water. To overcome this deficiency, materials such as silica oxide (SiO_2_) and MIP are used to modify the MNP. Magnetic molecular imprinted polymers (MIPs) consist of magnetic materials and non-magnetic polymers with the combined effect of their properties namely, selective recognition and magnetic separation [42].

In magnetic separation, Fe_3_O_4_ nanoparticles are encapsulated or coated as iron and iron salts by co-precipitation wherein a magnetic material added to a suspension containing a template. Modified components such as PEG, SiO_2_ are able to prevent flocculation of Fe_3_O_4_ nanoparticles. The most common and simplest fabrication technique is bulk polymerization in which the reaction takes place in a small amount of solvent to precipitate as an imprinted polymer. However, during polymerization, the components form agglomerates and reach irregular sizes which can damage the binding sites. Therefore, MIPs are subjected to post-treatment processes including, crushing, milling, and sieving to avoid this agglomeration. However, this rigorous process demands a long reaction time which provides only 30–40% of polymer recovery. In addition, to compare the selectivity against the targeted template, a non-imprinted magnetic preparation (MNIP) was carried out by following all process steps but without adding template molecules. MNIP also exhibits a strong but nonspecific binding capacity due to the interaction between the template and the polymer [42].

**Figure 1:**
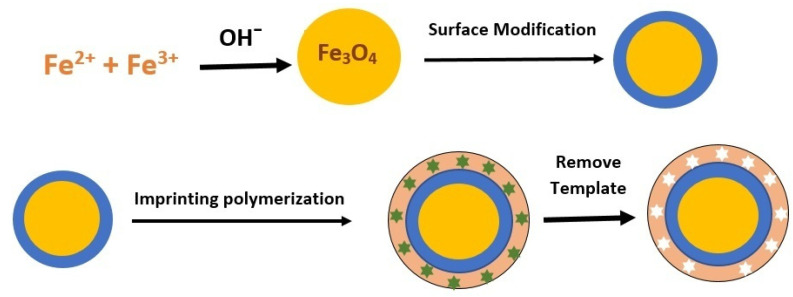
The general steps in the preparation of magnetic molecularly imprinted polymer.

Compared to conventional MIPs, MMIPs exhibit many superior characteristics involving fast and effective binding to the target analyte, and a shorter pre-treatment time [7]. The sorbent does not need to be packed into an SPE cartridge as in traditional SPE, and phase separation can be easily produced by applying an external magnetic field [8]. The MMIP is made using a combination of magnets and MIP [43]. The general MMIP preparation steps using Fe_3_O_4_ can be seen in Figure 1.

### 2.1. Magnetic Core-Shell Synthesis

Magnetic solids have two main applications in analytical chemistry, namely, the purification or separation of chemical samples (especially magnetic-SPE) and the use of biosensors or sensors, applications that are currently gaining popularity. Magnetic particles were initially applied to separate biological species and have been applied for decades to improve the separation of chemical species with various properties. An important aspect of magnetic particles is the method used for their synthesis, as their composition determines their compatibility and suitability for a particular application. Fe, Ni and Co are three well-known ferromagnetic metal elements in the periodic table. There are various magnetic materials involving metals, metal oxides, metal alloys and ferrites that are based on simple ferromagnetic elements [13]. Several types of magnetic materials are used in sample preparation, including nickel [44], Hematite iron (III) oxide (γ-Fe_2_O_3_) used in several MMIP synthesis [45,46,47]. In Can et al.’s [48] study, namely the comparative of nanosized iron oxide particles, magnetite (Fe_3_O_4_), maghemite (γ-Fe_2_O_3_) and hematite (α-Fe_2_O_3_), using ferromagnetic resonance showed a Fe_3_O_4_ particle size of 23.0 ± 0.6 nm, maghemite (γ-Fe_2_O_3_) 25.5 ± 0.5 nm and hematite (α-Fe_2_O_3_) 54 ± 5, indicating that the particle size of γ-Fe_2_O_3_ is smaller than α-Fe_2_O_3_ and the value of magnetization saturation (MS) using VSM is Fe_3_O_4_ 12.4 emu/g, γ-Fe_2_O_3_ 9.1 emu/g and α-Fe_2_O_3_ 1.3 emu/g indicates a value MS γ-Fe_2_O_3_ is bigger than α-Fe_2_O_3_ Fe_3_O_4_ [19,49,50] and nickel (II) oxide (NiO) [51]. However, the most commonly used magnetic material is Fe_3_O_4_ because of its easy fabrication, low toxicity and, most importantly, its abundant hydroxyl surface, which allows for further modification processes to be easily carried out [52]. Fe_3_O_4_ NPs can be easily synthesised by co-precipitation [52,53,54] and can also be prepared using the solvothermal method [26,55]. Fe_3_O_4_ NPs are usually coated with oleic acid before being further modified, to produce a better dispersion [49].

A summary of the MMIP method using Fe, Ni and Co magnetic particles can be seen in Table 1.

#### 2.1.1. Fe_3_O_4_

Fe_3_O_4_ is an easily prepared substrate with low toxicity, good biocompatibility, fast magnetic susceptibility and high surface area, and is the most commonly used support [7]. Magnetite Fe_3_O_4_ was prepared by the co-precipitation method, from a mixture of 0.01 mol FeCl_2_·4H_2_O and 0.02 mol FeCl_3_·6H_2_O dissolved in 100 mL water. The mixture was stirred vigorously and cleaned with nitrogen gas, then a solution of sodium hydroxide [8,56] or ammonia (NH_4_OH) [57] was added. After one hour, the magnet was isolated from the solvent using an external magnet and washed several times with water [8,56].

Several studies involve the MMIP polymerisation process using Fe_3_O_4_ as the magnetic core. In the study by Ali Zulfikar et al. [56], a poly vinyl chloride (PVC) MMIP sample solution was successfully synthesised for the selective separation of di(2-ethylhexyl)phthalate (DEHP) using Fe_3_O_4_ as the magnetic core. A magnetisation saturation (MS) value of 39.92 emu/g was produced using a vibrating sample magnetometer (VSM), indicating that the MMIP is superparamagnetic (SPM), and the resulting MMIP was better than the MNIP, with an imprinting factor (IF) value of 3.37, a maximum adsorption capacity value of 17.21 mg/g and a recovery percentage of around 91.03–99.68%.

In the study by Chen et al. [25], Fe_3_O_4_@SiO_2_–MPS was used as a sorbent in the magnetic SPE of resveratrol in wine (where MPS is 3-(trimethoxysilyl) propyl methacrylate). The MMIP showed a high MS capability of 53.14 emu/g, leading to fast separation, a high adsorption capacity capability for resveratrol and contained homogeneous binding sites. The recovery of spiked samples ranged from 79.3% to 90.6%, with a limit of detection (LOD) of 4.42 ng/mL. In the study by Fu et al. [58], Fe_3_O_4_ cyclodextrin material (Fe_3_O_4_-CD) was used for the rapid and specific adsorption of zearalenone. The results of the test of the magnetic properties of Fe_3_O_4_ NPs showed SPM properties; the coercivity and residual magnetic field strength were close to zero, and the saturation magnetic field strength was 99.68 emu/g for Fe_3_O_4_, 42.81 emu/g for the MMIP and 38.10 emu/g for the MMIP–CD. In real sample testing, the limit of quantification (LOQ) and LOD were 0.1 ng/kg and 0.3 ng/kg, respectively.

In the study by Habibi et al. [59], the highly lipophilic drug buprenorphine was analysed in human urine samples using an Fe_3_O_4_ magnetite core surrounded by polyamidoamine and buprenorphine as a template. The magnetic properties results using a VSM showed supermagnetic properties, and the MS of Fe_3_O_4_-oleic acid and MMIP nanoparticles (MMIPNP) were 55.75 and 59.04 emu/g, respectively. The relative recovery was 97.4–100.3%, and the LOD and LOQ were 0.21 and 0.71 ng/mL, respectively. The extraction of herbicide chloroacetamide from environmental water samples was carried out using the amphiphilic MMIP method with Fe_3_O_4_ microspheres [9]. Under optimized conditions, good linearity (0.1–200 g/L) and good precision (relative standard deviation (RSD) < 7%) were demonstrated, with a low detection limit (0.03–0.06 g/L), and recovery ranged from 82.1% to 102.9%.

Tadalafil analysis on the surface of MNPs was carried out by Li et al. using Fe_3_O_4_@SiO_2_ [16]. VSM analysis showed MS values of 61 and 42 emu/g for Fe_3_O_4_@SiO_2_ and MIP-coated MIP, respectively, and a recovery value in the range of 87.36 to 90.93%, with RSD < 6.55%. Purification of alkaloid isomers (theobromine and theophylline) from green tea using magnetic solid phase extraction (MSPE) with Fe_3_O_4_ as the core [60] showed the practical recovery of theobromine and theophylline in green tea was 92.27% and 87.51%, respectively.

The MMIP polymer synthesised by SPE for the efficient separation of racemic tryptophan (Trp) in aqueous media used Fe_3_O_4_ (Fe_3_O_4_@MIPs). The magnetic properties of Fe_3_O_4_-NH_2_ and Fe_3_O_4_@MIPs were measured by VSM and showed a MS of 75 and 69 emu/g, respectively, indicating a high level of superparamagnetism. The respective maximum adsorption capacity values for L-Trp and D-Trp were 17.2 ± 0.34 mg/g and 7.2 ± 0.19 mg/g, and good selectivity to L-Trp was observed, with an IF of 5.6 [10]. Another MMIP was synthesised by Qin et al. for the adsorption of sulphonamides using a surface imprinting technology with Fe_3_O_4_-chitosan (Fe_3_O_4_-CS) as a template for a mixture of sulphamethazine (SMZ) and sulphamethoxazole (SMX) molecules [61]. The magnetic property test showed the presence of symmetry at the origin and coercivity, and a resonance was zero. The MS values were 69.94, 20.84 and 3.91 emu/g, indicating SPM properties. Maximum adsorption (Q) capacity values were Q (SMX) = 4.32 mg/g and Q (SMZ) = 4.13 mg/g, and recovery and RSD were from 85.02 to 102, respectively, 98% and from 2.77 to 6.47%.

The development of methods in the synthesis of magnetite Fe_3_O_4_ has been carried out. Recent research conducted by Ferrone et al. [62] carried out the simple synthesis of Fe_3_O_4_@-activated carbon from wastepaper for dispersive magnetic solid-phase extraction of Non-Steroidal Anti-Inflammatory Drugs (NSAIDs) in human plasma. The wastepaper showed an excellent capacity to absorb the iron oxide by forming a colloidal solution simply due to cellulose, which entrapped iron in its fibrous structure. The SEM images show the morphology of the samples after grinding, all of which appeared very similar, made of large particles (tens of microns) heterogeneously distributed. The XRD patterns showed a lower crystallinity of the Fe_3_O_4_ phase, this could be due to the sluggish kinetics of the formation of magnetite, considering that the iron precursor was likely entrapped in the cellulose fibers and less exposed to the nitrogen atmosphere. The method developed herein proved to be fast and accurate.

##### 2.1.2. γ-Fe_2_O_3_

γ-Fe_2_O_3_ NPs were used by Abdel-Haleem et al. as magnetite particles in electrochemical sensors, showing unique properties in terms of increasing sensor sensitivity, increasing the LOD and shortening the analysis time compared to non-magnetic NPs [63,64]. Iron oxide @ carbon nanotubes (Fe_2_O_3_@MWCNTs) and MIP nanocomposites were synthesised during the manufacture of carbon paste electrodes for the potentiometric detection of ivabradine hydrochloride in biological and pharmaceutical samples. The result showed low magnetic properties and MS values reaching 0.05 emu/g and 1.4 emu/g of γ-Fe_2_O_3_ tested by VSM for MWCNTs and Fe_2_O_3_@MWCNTs, respectively. This study demonstrated low magnetisation values for Fe_2_O_3_@MWCNTs compared to carbon nanotubes (MWCNTs), which they attributed to the low Fe_2_O_3_ content of around 0.55 wt%, established using X-ray Fluorescence (XRF), but demonstrated a highly sensitive and selective carbon paste sensor for the potentiometric determination of ivabradine hydrochloride in physiological fluids.

#### 2.1.3. Nickel (Ni)

Magnetic nickel and magnetic nickel (II) oxide (NiO) NPs have also been used for the preparation of electrochemical and MIP sensors. In the study by Li et al. [51], NiO MNPs were coated with MIP, using chlortoluron as a template. The study showed a NiO magnetic hysteresis loop (100 nm) of 66.7 emu/g, and the curve results showed that the NiO NPs had high magnetic activity, ferromagnetism and paramagnetism.

#### 2.1.4. Cobalt (Co)

Research conducted by Wu et al. [65] used magnetic cobalt nanoporous carbon (Co-MNPC) as an alternative to Fe_3_O_4_ cores in the preparation of magnetic MIPs (Co-MNPC@MIPs) of zeolitic imidazolate framework-67 (ZIF-67). The results showed a coarse surface structure of Co-MNPC@MIPs, which implied that the porous structure of the MIP shell could interact with the target molecule. The MS of the Co-MNPC was 45.07 emu/g, which decreased to 34.55 emu/g for the Co-MNPC@MIPs after the formation of the MIP shell, although the magnetic nano adsorbent still had high magnetism.

The summary in Table 1 shows that the MS values of the MMIPs are different when different magnetic particle are used. A decrease in the MS value of MMIPs compared to Fe_3_O_4_ may be caused by the formation of a magnetically inactive layer containing spins that are not collinear with the magnetic field [66]. However, even though the MS of the MMIP is substantially reduced, the material remains magnetic enough to act as an effective magnetic separation carrier [31].

**Table 1:** Summary of the MMIP methods using Fe, Ni and Co magnetic particles.

Analyte	Magnetic Particle	Magnetisation Saturation	Magnetic Activity	Ref.
Di(2-ethylhexyl)phthalate (DEHP)	Fe_3_O_4_	39.92 emu/g	Superparamagnetic	[56]
Resveratrol	Fe_3_O_4_	53.14 emu/g	Superparamagnetic	[25]
Buprenorphine	Fe_3_O_4_	59.04 emu/g	Supermagnetic	[59]
Tadalafil	Fe_3_O_4_	42 emu/g	Superparamagnetic	[16]
Zearalenone	Fe_3_O_4_	38.10 emu/g	Superparamagnetic	[58]
Enantiomer tryptophan (Trp)	Fe_3_O_4_	69 emu/g	Superparamagnetic	[10]
Sulphonamides	Fe_3_O_4_-chitosan	3.91 emu/g	Superparamagnetic	[18]
Ivabradine	Fe_2_O_3_	1.4 emu/g	Low magnetic properties	[64]
Chlortoluron	Nickel (II) oxide (NiO) magnetic nanoparticles	66.7 emu/g	High magnetic activity, ferromagnetism and paramagnetism	[51]
Zeolitic Imidazolate Framework-67 (ZIF-67)	Cobalt nanoporous carbon (Co-MNPC)	34.55 emu/g	High magnetism	[65]

Table 1 shows that Fe_3_O_4_ is the most widely used magnetic component, showing a higher MS value than Ni and Co magnetic particles, with the highest value of 69 emu/g. Marfà et al. indicated that magnetite (Fe_3_O_4_) is the most widely used due to its biocompatibility, strong superparamagnetism, good catalytic activity and simple preparation procedure [67]. Other reasons include its low toxicity, good biocompatibility, fast magnetic susceptibility and high surface area, making it the most commonly used support [7].

According to Nguyen et al., Fe_3_O_4_ NPs exhibit SPM or ferrimagnetic (FM) behaviour. In the presence of an external magnetic field, the magnetic material reaches a saturation magnetisation value (MS), and SPM NPs have several advantages, such as preventing NP agglomeration (caused by magnetic attraction) and having a sensitive response to a remote-controlled magnetic field. In contrast, FM materials exhibit certain magnetisation values in the absence of an external magnetic field. Therefore, FM NPs always retain strong magnetic properties, which is potentially useful for applications where strong magnetic properties are always required. Fe_3_O_4_ is more widely used than iron oxide or other ferrite spinel oxides (Co, Ni, Mg, etc.) because of its superior magnetic properties [68].

Primary iron oxide MNPs easily oxidize in air and tend to aggregate into large groups. To prevent this, the MNPs are coated with stabilisers, such as silica and polymers; conversely, they can be embedded in a chemically inert protective matrix. In general, Fe_3_O_4_ particles are initially encapsulated with tetraethyl orthosilicate (TEOS) via a typical sol-gel reaction, leading to Fe_3_O_4_@SiO_2_ hybrid particles, oleic acid [8] and chitosan [31]. Fe_3_O_4_@SiO_2_ is more commonly used because the SiO_2_ layer protects the core from oxidation or dissolution in the following reactions. In addition, the silica shell minimizes the formation of large clusters and improves MNP biocompatibility. The silanol groups on the silica surface thus provide surface functionalization for further polymerisation. It is important to underline that although the silica shell can decrease Fe_3_O_4_ magnetisation, its magnetic properties are still sufficient for further applications [69].

### 2.2. MMIP Polymerisation

Based on the synthesis process, MMIP components consist of magnetic particles, magnetic surface modification components and polymerisation components, such as templates, functional monomers, cross-linkers and porogens [8,25,31,43,56,70]. MSPE primarily involves a magnetic adsorbent of MNPs, and the target analyte bound to the magnetic adsorbent by chemical or physical interaction, after which the complex is removed from the sample solution by an external magnetic field [70]. Since MMIPs are composed of magnetic material (Fe_3_O_4_ NP) and MIP, and the MIP is coated with Fe_3_O_4_ NP, the core-shell magnetic material not only has magnetic properties, but also exhibits high selectivity for the target analyte [28].

A good MIP layer will produce a sorbent material with selective recognition capacity of the target analyte; therefore, determination of the MIP layer design is very important during MMIP fabrication [43]. A number of MIT strategies are based on two traditional polymerisation principles, which are FRP [11,18] and sol-gel polymerisation [32,71]. Successful molecular imprinting must apply strong and specific bonds between templates and functional monomers. Several types of magnetic composites with high specific surface area were developed and served as printing supports to obtain a good performing MIP coating with more accessible bonding sites [43]. A comparison of the analytical features of the developed MMIP method with previously reported methods using FRP and sol gel techniques is provided in Table 2.

#### 2.2.1. Free Radical Polymerisation (FRP)

The most popular, most frequently used and well-developed synthesis method in the MIP preparation process is FRP [43]. The FRP preparation methods are: suspension polymerisation [72], emulsion polymerisation [73] and precipitation polymerisation [74]. In the successful manufacture of MMIP, the active group (carbon-carbon double bond) is first grafted onto the MNP for better surface immobilization. An initiator is added, and FRP is then initiated between the surface graft active groups with the addition of the monomer, which is crosslinked under vigorous stirring and the application of N_2_ gas. Acrylic-based MIP is prepared by radical polymerisation. Different functional monomer designs form donor-receptor complexes with specific templates, from organic molecules to inorganic molecules [75,76]. Methacrylic acid is the most commonly used functional monomer due to it being more flexible in FRP [43] and possessing good specific selectivity [77].

#### 2.2.2. Sol-Gel Polymerisation

The sol-gel technique involves the hydrolysis, polymerisation, gelation, aging and heat treatment of inorganic substances or metal alkoxides. The combination of molecular printing with sol-gel technology generates an inorganic network structure, which forms a rigid organic-inorganic hybrid sol-gel material [78]. The sol-gel method is based on silica and inorganic-organic hybrid materials using organically modified silica. In the process, the template and functional monomers are combined through noncovalent interactions, by hydrogen bonding, hydrophobic π-π interaction, etc. The significant advantages of the sol-gel technique are the easy preparation, gelation treatment at room temperature, and high porosity and surface area [79].

Li et al. carried out SPE for MMIP-based norfloxacin using a sol-gel polymer, with a bifunctional monomer giving the highest adsorption capacity (312.08 g/mg) and the best selection factor (5.41) [80]. The bifunctional monomer had the best extraction ability, was successfully applied to the extraction of norfloxacin in lake water and showed good accuracy and precision. MIP silica sol-gel is very widely used, having the advantages of simple fabrication, an environmental friendly solvent (aqueous solution) and mild conditions [78]. In addition, the sol-gel technology is able to produce three-dimensional silicate networks with high porosity in a simple way, with the ability to form excellent rigid physical properties due to the highly cross-linked silica structure, resulting in fine mould sites with high selectivity potential [81]. MIP silica sol-gel produces a strong matrix for a wide range of applications and exhibits minimal swelling in the presence of solvent, as well as maintaining the shape and size of the mould cavity. Silica is also highly compatible with aqueous and biological systems and is able to successfully encapsulate enzymes and antibodies without impairing their activity [78].

**Table 2:** Comparison of the analytical features of developed MMIP methods with previously reported methods using free radical polymerisation (FRP) and sol gel polymerisation.

Analyte	Magnetic Particle	Analytical Application	Synthesis Method	Q MMIP (µmol/g)	Q MNIP (µmol/g)	Recovery MMIP (%)	Ref.
Chloramphenicol	Fe_3_O_4_ magnetite	Honey	Suspension polymerisation	17.1	8.8	84.3–90.9	[8]
Resveratrol	Fe_3_O_4_@SiO_2_–MPS nanoparticles	Wine	Surface molecular imprinting	23.36	9.3	79.3–90.6	[25]
Tricyclazole	Chitosan Fe_3_O_4_	Rice and water samples	Precipitation Polymerisation	240.199	139.06	89.4 (rice), 90.9 (water)	[31]
Chloramphenicol	Fe(NO_3_)_3_·9H_2_O	Aquatic environment	Precipitation polymerisation	71.77, 107.0 and 120.8 at 298, 308 and 318 K	53.10, 71.44 and 87.14 at 298, 308 and 318 K.	-	[74]
Norfloxacin	Fe_3_O_4_@SiO_2_	lake waste water	sol-gel polymerisation	1301	1121	85.4–96.4	[80]
Imidacloprid	Fe_3_O_4_ magnetite	Water and apple samples	Suspension polymerisation	0.094	0.039	94.0–98.0	[82]

In order to produce selective analytical methods, various MIT methods were proposed, and MMIP magnetic-based methods were developed. FRP is the most widely used MIT (Table 2) because of its simple fabrication and wide choice of functional monomers [83]. The MMIP method with precipitation polymerisation is limited in its use due to accurate reaction conditions during the FRP process. Suspension polymerisation is a simple method which is suitable for the manufacture of porous MMIPs with spherical or particle morphology, since the Fe_3_O_4_ magnetic particles do not require functionalization [83]. However, it has the drawbacks of uncontrolled radical reactions and irregular morphological characteristics, and the passage of the inner binding site may be blocked, so that the number of extracted compounds will be limited. Sol gel-silica has an advantage as a part of sol-gel polymerisation as the resulting MIP will be compatible with water and has a simple and lightweight synthesis procedure [84]. The characteristics of the rigid polymer structure and high cross-linking provide good stability for sol-gel MIPs, but they also have faster mass transfer [20].

In the review, Poonia et al. mentioned the potentials and major drawbacks of various imprinting methods used for fabrication of MMIPs. In this review, the bulk polymerization synthesis has the advantages of being fast and easy to synthesize, does not require additional solvents or sophisticated instruments and is low cost but has disadvantages. Disadvantages include: long processing time due to grinding and sieving processes, low binding site affinity, low binding site capability during template removal, large particle size and low molding capability [42].

##### MMIP on Drug

During the real sample extraction of drug compounds (mainly in aqueous systems), water molecules will interfere with the rebinding between target analytes and the MIP, so stronger interactions are always desired. Table 2 shows the use of different sorbents according to the template [43]. Li et al. [80] synthesized MMIP-based norfloxacin using a sol-gel polymer, with a bi-functional monomer, Aminopropyltriethoxysilane (3-APTES) and Methacryloxypropyltrimethoxysilane (MTEOS) used as monomers and tetramethyl orthosilicate (TEOS) as cross linker through a one-pot sol-gel polymerization. Showed highest adsorption capacity (312.08 g/mg) and the best selection factor (5.41). The bifunctional monomer had the best extraction ability, was successfully applied to the extraction of norfloxacin in lake water and showed good accuracy and precision. In research Laskar et al. [31] synthesized MMIP using tricyclazole/Fe_3_O_4_ chitosan with non-covalent binding polymerization involving methacrylic acid (MAA) as functional monomer, divinylbenzene (DVB-80) as crosslinker, 2,2’ -azobisisobutyronitrile as initiator, exhibiting a maximum binding capacity of 4579.9 g/g, a reusable imprinted polymer with high selectivity and specificity properties can be utilized as an adsorbent for solid-phase extraction in sample preparation for tricyclazole residue analysis in complex environmental matrices.

##### MMIP on Macromolecules

Proteins such as antibodies and enzymes are usually used as elements of recognition for the diagnosis and treatment of disease. The imprinting of macromolecules such as proteins is still a challenge. First, the protein is insoluble or easily deactivated in commonly used printing solvents. Second, the protein conformation is very flexible, which can cause changes during polymerization, so that the final binding site may not match the target analyte with the original structure. In addition, the large size of the protein makes it difficult to remove from the 3D crosslinking polymer, and the binding sites away from the surface are also inaccessible [42].

Surface imprinting is one of the most efficient strategies to ensure the accessibility of binding sites during protein extraction. In a recent work presented by Liu et al. [85], the initiator was grafted onto the surface of an amino-functioning Fe_3_O_4_ nanoparticle, on which a water-compatible layer was grown. Core-shell MMIP successfully extracted deoxyribonuclease I (31 kDa) in complex biological samples without reducing its activity. Combining DSPE with common fluorescent probe detection yields a linear working range of 10–300 ng mL^−1^ for the obtained deoxyribonuclease I.

The preparation and introduction of MMIP on macromolecules has also been carried out [86,87]. Kan et al. [86] synthesized MMIP for protein recognition. MMIP was synthesized by copolymerization of γ-aminopropyltrimethoxysilane and tetraethyl orthosilicate on the Fe_3_O_4_ nanosphere surface, which is directly covalently bound to the bovine hemoglobin (BHb) template molecule. The value of adsorption capacity (Q) for MMIP 10.52 mg/g and MNIP 2.28 mg/g. MMIP exhibited fast adsorption dynamics, excellent specialized adsorption and recognition capacity for BHb. Jing et al. [87] synthesized MMIP for recognition of lysozyme n human serum sample, MMIP had high Q value 0.11 mg/mg^−1^ with a recovery of 92.5 to 113.7%.

##### MMIP Method Development

MMIP as a sensor has also been developed and shows advantages over traditional techniques such as chemistry and bio sensing because it has various disadvantages including lack of signal expression ability, longer response time with lower selectivity, and easy denaturation [42]. The loading of the MIP layer on the nanocomposite surface, together with the incorporation of the fluorescent sensor material, converts the active binding sites into a readable signal. Similarly, a new fluorescence sensing strategy for detecting 4-nitrophenol (4-NP) in food samples was developed by Zhu et al., using an MMIP sensor which exhibits dual recognition capability. Considering the analytical performance of the sensor, the observed detection limit was 23.45 nmol L^−1^ with a high imprint factor (12.2). The MMIP sensor exhibits a fast response time (2 min), confirming the dual recognition capability and uniform distribution of recognition sites. High recovery (93.20% to 102.15%) of the 4-NPs was observed due to the tendency of magnetic responsiveness and repeated reuse of the sensor showing minimal changes in fluorescence [88].

Terephthalic acid (TPA), which has been widely used as a precursor in the formation of polyester polymers (PET), was studied and used as a monomer in the innovative synthesis of new adsorbent materials by molecular recognition. Da Silva et al. first synthesized a new magnetically imprinted polymer (MMIP) using terephthalic acid as a functional monomer to extract atenolol (ATL) from human plasma by magnetic solid phase extraction (MSPE). The separation of ATL enantiomers was carried out by capillary electrophoresis using carboxymethyl-β-cyclodextrin (CM-β-CD: 5.5 mg) as a chiral selector on a background electrolyte with 125 mmol L^−1^ triethylamine pH 6.0 using a capillary with an inner diameter of 75 m. The resulting percentage recovery/relative standard deviation were for (−)–(S)-ATL 75.8 ± 6.3% and (+)–(R)-ATL 76.1 ± 5.7%, respectively. MMIP imprinting test confirmed that the material was selective for ATL, with low recoveries for other drugs [89].

The synthesis of MMIP was carried out in three stages: the manufacturing of magnetic core particles, the magnetic coating of the core-shell using modified components, and the synthesis of MMIP using polymerisation components. The factors that affect the production of the desired MMIP at each stage of the process are discussed in the following section.

## 3. Factors Affecting MMIP Synthesis

### 3.1. Factors Affecting the Manufacture of Magnetic Core Particles

In the manufacturing of magnetic core particles to meet the analysis requirements of different target analytes in different samples, various MMIP structures are generated to produce selective MMIPs [43]. Depending on the field of application, various types of core-shell structure can be synthesised, such as the Janus-type, dumbbell, shell-core-shell, yellow-shell, matrix-scattered and core-shell [90]. The core-shell structure is the most widely used and involves the magnetic phase as the core and the polymer phase acting as the shell [41]. It is widely used due to its magnetic properties, biocompatibility, excellent surface-to-volume ratio and high binding capacity [90]. In the core-shell structure, the polymer coating prevents the core from oxidizing and aggregating but weakens the magnetic performance at the same time [43]. What needs to be considered in the manufacturing of magnetic core particles is the morphological characterization, size and size distribution of the prepared product [33]. Various techniques are available to make magnetite (Fe_3_O_4_), as the core, using co-precipitation, the solvothermal/hydrothermal method, oxidation, injection flow synthesis, the supercritical fluid method, microemulsion, thermal decomposition, chemical vapour deposition, electron beam lithography, microwave assistance and sonochemistry [39].

The most commonly used techniques for the magnetic preparation of MMIP NPs are the co-precipitation and solvothermal/hydrothermal techniques [12,90]. The first step in the manufacturing of MMIP is to make magnetite, with the final product producing iron (II, III) oxide or ferrosoferric oxide (Fe_3_O_4_) [36]. Magnetite is obtained by co-precipitation, which consists of a mixture of hydrated iron (II) chloride (FeCl_2_·H_2_O) and iron (III) chloride (FeCl_3_·6H_2_O), and can also be obtained from iron(II) sulphate (FeSO_4_·7H_2_O). Magnetite synthesized using FeCl_3_·6H_2_O and FeCl_2_·H_2_O has higher magnetic properties, namely 55.4 emu/g compared to that synthesized using Fe_2_(SO_4_)3·nH2O and FeSO_4_·7H_2_O have magnetic properties of 46.7 emu/g [91]. Furthermore, both reactions were carried out in a solution of sodium hydroxide (NaOH) or ammonia (NH_4_OH) at a temperature range of 80–100 °C [92,93,94]. Magnetite synthesised using NH_4_OH solution as a precipitate [95] had a higher crystallinity than that synthesised using NaOH solution [96].

Shao et al. performed magnetic particle synthesis using a solvothermal method, which involved dissolving FeCl_3_.6H_2_O and sodium acetate in ethylene glycol with vigorous stirring, resulting in a yellow homogeneous solution, which was then transferred to an autoclave, sealed, heated at 200 °C for 8 h, and then cooled to room temperature [56]. The reaction product was black magnetite particles, which were then washed several times with ethanol and dried at 60 °C for 12 h [55].

The solvothermal method has the advantage of increasing the effective collision of metal ions by accelerating the fast convection of the solvent and the active diffusion of the solute in the solvothermal state, for the formation of NPs with a narrow size distribution, resulting in a more uniform size and better dispersion properties [97,98]. The factors that must be considered in this method are the type of iron source, solvent, amount of the iron source, temperature and time, as they affect the quality of the final product. However, this method involves higher costs and a greater effort due to the very high temperatures involved in the heating step [98].

The advantage of the co-precipitation method is that a large number of NPs can be synthesised, and it is also water-soluble, biocompatible with iron oxide NPs and an easy procedure [97]. However, its weakness is that the resulting particle size is irregular as control of the particle size distribution is limited, because only kinetic factors control it. Another weakness of this method is the broad distributions of sizes and the aggregation of particles [97,98].

The résumé of advantages and disadvantages of techniques for the magnetic preparation of MMIP are given in Table 3.

#### 3.1.1. Effect of Temperature and Reaction Time in the Manufacture of Magnetite Fe_3_O_4_

Magnetic properties are highly dependent on size. To obtain SPM Fe_3_O_4_ NPs with adjustable size, the size of the iron oxide NPs has to be controlled during synthesis by changing the reaction temperature [99] and reaction time [36,68,100]. This procedure can easily control the size of the NPs and prepare large quantities of particles, but at the same time, a higher reaction temperature will change the crystal structure [99].

Gao et al. [36] initially used a temperature of 220 °C for 2 h, resulting in an irregular shape and wide particle size distribution on the Fe_3_O_4_ scanning electron microscope (SEM) results. When the temperature was increased to 240 °C for 2 h, the product showed coarse spherical particles and a size of 6.5 nm with good monodispersity; while the size distribution was concentrated in the range of 5–8 nm when the temperature was maintained at 260 °C for 2 h, with the size and morphology of the product tending to be more uniform and regular. The researcher therefore concluded that high temperature produces a more uniform form of magnetite Fe_3_O_4_ with relatively spherical particles. The production of more uniformly sized Fe_3_O_4_ particles seems to indicate that a high reaction temperature will be required for the formation of homogeneous MNPs. This is attributed to the high temperature of 260 °C, allowing for a sufficient reaction rate, while the low temperature decreases the reaction rate and the diffusion of active species, which expands the size distribution and induces disproportionation and aggregation.

Gao et al. [36] also researched the effect of reaction time on the formation of Fe_3_O_4_ NPs and found that the reaction time significantly affected the size of the NPs produced. Transmission electron microscopy (TEM) images of the NPs obtained at 260 °C with different reaction times showed that the size of the Fe_3_O_4_ NPs gradually increased as the reaction time extended. After a reaction time of 6 h, monodispersed Fe_3_O_4_ NPs with a narrow size distribution were obtained, with the average diameter increasing from 10.5 nm to 12 nm when the reaction time was extended to 12 h. This shows that the size of Fe_3_O_4_ NPs increases linearly with high temperature and reaction time.

In the research by Nakaya et al. [99], the synthesis of Fe_3_O_4_ magnetite monodispersed NPs was also performed to observe the effect of temperature and reaction time on the particle size. The effect of the reaction temperature on the particle size was determined by TEM images of the synthesised NPs, with the particle size tending to increase with increasing reaction temperature: when the reaction temperature was 200 °C, the resulting NPs showed a spherical shape, with a particle size of 5.3 ± 0.6 nm; when the reaction temperature was increased to 250 °C, 280 °C and 300 °C, the spherical particle sizes increased to 8.2 ± 0.6 nm, 13.0 ± 0.9 nm and 20.4 ± 2.2 nm, respectively. The effect of reaction time on the particle size and structure was also analysed using TEM images of synthesised Fe_3_O_4_ NPs as a function of the reaction time. The reaction temperature was set at 280 °C for 1, 3 and 6 h. The mean diameters of the resulting NPs were 6.6 ± 1.0 nm, 13.0 ± 0.9 nm and 19.5 ± 1.7 nm after 1 h, 3 h and 6 h, respectively, showing that a longer reaction time causes an increase in particle size with a narrow size distribution. However, when the reaction time was longer than 6 h, no nanoparticles larger than 20 nm were obtained.

In the study by Maity et al. [100], the MS of the magnetite particles increased due to the higher reaction temperature and reaction time, and the particle size and distribution were also affected. This study investigated the effect of surfactants or solvents on the effects of temperature and time to produce magnetite NPs with high MS values, while maintaining smaller sizes in an acceptable size distribution. The study used temperatures of 220, 265, 300 and 330 °C, respectively, at a reaction time of 2 h. The X-ray diffraction (XRD) pattern showed that the peak width of the Fe_3_O_4_ phase decreased with the increasing reaction temperature due to an increase in particle size or particle crystallinity. The average crystal sizes for the samples were 4.9, 5.8, 9.4 and 14.3 nm, respectively. This shows that the particle size increases with increasing reaction temperature, but uncontrolled crystal growth occurs at higher reaction temperatures, and the MS value increases from 46 to 74 emu/g when the reaction temperature is increased from 220 to 330 °C. The effect of a 0.5 and 4 h reaction time at 300 °C was investigated using TEM images, which showed that the mean particle size increased from 7 to 12 nm as the reaction time increased from 0.5 to 4 h, while the particle size distribution widened and the MS value increased from 57 to 65 emu/g. The increase in MS with the increasing reaction time could also be due to an increase in particle size or particle crystallinity [100]. A temperature of 300 °C with a time of 0.5 and 4 h resulted in a very narrow size distribution and an increase in the value of MS [100]. Gao et al. [36] said that high temperature at any given time will increases the rate of reaction,, whereas low temperature decreases reaction rate and diffusion of active species, which expands the size distribution and induces disproportionation and aggregation. Several research studies on the effect of temperature and reaction time in the manufacturing of magnetite Fe_3_O_4_ have shown that the size of the Fe_3_O_4_ NPs increases linearly with high temperature and reaction time [36,99], and that the MS of the magnetite particles also increases due to the higher reaction temperature and reaction time [100], as shown in Table 4.

#### 3.1.2. Effect of pH Value

The pH has an effect on the synthesis of Fe_3_O_4_. Increasing the pH value will increase the amount of Fe(OH)_3_ and Fe(OH)_2_ due to an increase in the hydrolysis process of Fe^3+^ and Fe^2+^, thereby increasing the amount of Fe_3_O_4_ [101]. The pH of the [OH^−^] concentration was used to control the nucleation and growth of Fe_3_O_4_ NPs and to influence the particle and magnetic properties [102].

The study by Faiyas et al. [35] proved that the higher the pH (pH 11 with the addition of merchaptoethanol), the purer the synthesised particles and the smaller the crystal particle size. The sample at pH 6 (without the addition of merchaptoethanol) showed a particle size of 14.25 nm, while the sample at pH 9 (without the addition of merchaptoethanol) showed a particle size of 19.3 nm and the sample at pH 11 (with the addition of 5 mM merchaptoethanol) showed a particle size of 8.02 nm. The XRD pattern of the magnetite (Fe_3_O_4_) phase of the sample with a pH value of 11 shows no other phases, such as Fe(OH)_3_ or Fe_2_O_3_, which are by-products of the Fe_3_O_4_ precipitation procedure. The nanomagnetic synthesised Fe_3_O_4_ particles are very pure, and all of the samples were nanocrystalline in the presence of wide peaks. Sirivat et al. [57] also showed that the higher the pH (8–11), the smaller the particle size. The results of several research studies on the effect of pH show that the higher the pH, the smaller the particle size.

### 3.2. Factors Affecting the Magnetic Coating of the Shell Using Modified Components

#### 3.2.1. Effect of Modified Component Types

The modification of the MMIP surface was a factor affecting the production of MMIP with good water solubility, biocompatibility, dispersion stability and active functional groups [33]. Usually, the modification objective is achieved by introducing a protective layer on the MNP surface. Coating materials mainly include inorganic materials (silica, carbon, precious metals, etc.) and organic materials (surfactants, polymers, etc.) [12]. In addition, it is necessary to add a stabiliser, because the high iron precursor concentration of the magnetic component can lead to the formation of large amounts of seeds, leading to an increase in the yield of small NPs. When the ionic strength in the system shows a slowed growth and nucleation rate, it encourages the emergence of NPs with small sizes and can also avoid agglomeration. Stabilisers commonly used in modified co-precipitation methods include organic anion chelators (citric, glucose, oleic acid, etc.) and polymer surface complexing agents (chitosan, carboxylated chitosan, starch, polyethylene glycol (PEG), etc.) [12]. For better dispersion, Fe_3_O_4_ is coated with oleic acid before further modification. Silica-coated Fe_3_O_4_ (Fe_3_O_4_@SiO_2_) is also a common choice, because the SiO_2_ layer is a good medium for immobilizing different functional groups [43].

The characteristic solvent pores in bulk sol-gel silica produce a mixture of micro and mesoporous pores with a wide distribution of pore sizes and shapes. There are three categories of pore sizes based on their smallest diameter: micro pores having diameters smaller than 2 nm, macro pores having diameters greater than 50 nm, and pores having diameters between 2 and 50 nm. Irregularly shaped pores have the smallest diameter. Long conduits that are open at one or both ends are classified according to the diameter and not the length of the channel. The addition of a quaternary ammonium surfactant to the synthesis of a sol-gel molecular sieve resulted in a highly porous material with a long channel-shaped pore structure of uniform diameter, arranged in a two-dimensional hexagonal shape. The pore structure is formed by the surfactant, which forms a 2D hexagonal liquid crystal phase in solution [84].

##### Oleic Acid

**Figure 2:**
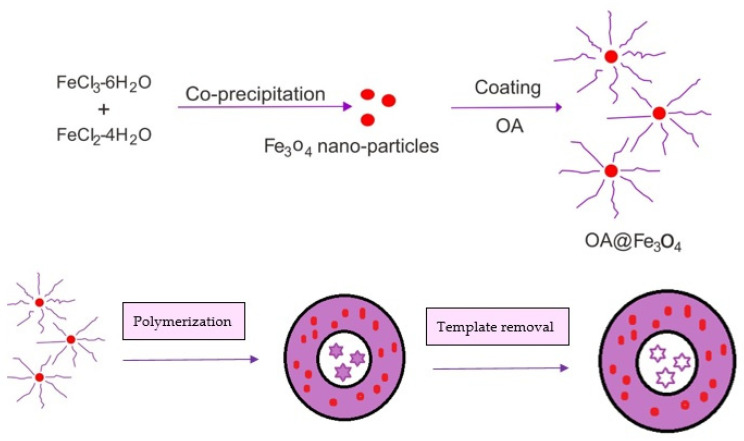
Scheme for the preparation of MMIPs with an oleic acid topcoat.

Oleic acid is used in high quantities in the surface modification of MMIP as a topcoat over the printed system, imparting the amphiphilic properties that make it compatible with water, as well as other solvents. Surface modification of MMIP with oleic acid is carried out because most MMIP is developed in organic solvents; thus, they often retain their selectivity in aqueous solvent systems, as well as in biological fluids, due to the presence of weaker electrostatic hydrogen bonds. Due to the presence of oleic acid on the surface of the MMIP, hydrogen bonds between the template and the polymer matrix are preserved from rapid destruction when in water [103,104,105]. The scheme for the preparation of MMIPs with an oleic acid topcoat can be seen in Figure 2.

The problem faced in the formation of Fe_3_O_4_ NPs is that nanoscale particles with a large surface-to-volume ratio will cause aggregation during particle formation, through van der Waals attraction between particles. To overcome this problem, a stabiliser is used, which can adhere to the particle surface and provide spatial isolation in the synthesis system [106].

Several MMIP studies using Fe_3_O_4_ particles coated with oleic acid have succeeded in producing a stable polymer surface and showing a good percentage recovery. An example is the analysis of chloramphenicol in honey samples by Chen et al. [8], which used the MMIP method in the extraction process, with Fe_3_O_4_ as a solid magnetite, and oleic acid and polyvinylpyrrolidone (PVP) as magnetic surface modification components, giving a Q max value of 5679 μg/g compared to MNIP with 2922 μg/g.

In the study by Ilktaç et al. [82], MMIP were used to, pre-concentrate, trace levels of imidacloprid in water and apple samples. Oleic acid was used as a magnetic surface modification component, resulting in recoveries in the range of 92.0–99.0%. Liu et al. [49] synthesised novel MMIPs for SPE for the selective separation of metronidazole in cosmetics, using oleic acid for the surface modification of Fe_3_O_4_ NPs, obtaining a Q value of 10,800 μg/g for the MMIP and 4920 μg/g for the MNIP.

MMIP NPs were generated by Attallah et al. [21] for the simultaneous extraction of 6-mercaptopurine (6-MP) and its active metabolite thioguanine (TG) in human plasma using Fe_3_O_4_@oleic acid, and showed that the Q MMIP 6-MP was 822.29 μg/g and the Q TG was 519.15 μg/g, higher than the MNIP with a Q 6-MP of 537.92 μg/g and a Q TG of 352.24 μg/g; the recovery was in the range of 8.89–103.03% for 6-MP and 85.94–98.27% for TG.

##### Chitosan

**Figure 3:**
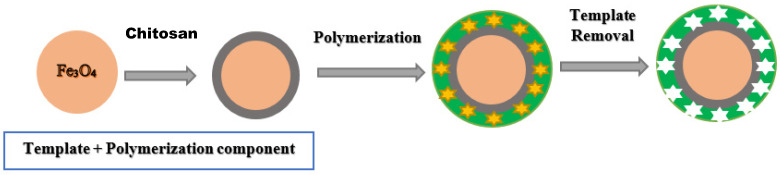
The preparation of MMIPs using chitosan.

Chitosan is a linear biopolymer, chitin derivative consisting of N-acetyl-d-glucosamine and d-glucosamine groups, linked by 1–4 bonds. It is present in the cell walls of several fungal strains, especially zygomycota, and is becoming attractive as a new functional material in various analytical, industrial, environmental and biomedical fields. The largest producers of chitosan are in Japan, India and Norway [107,108]. Chitosan is used for the preparation of MMIPs is depicted in Figure 3. MMIP made by combining the advantages of chitosan is expected to produce new and more profitable materials. Chitosan-based composites have emerged as promising materials with excellent thermal, mechanical, electrical and optical properties, which play an important role in the elaboration of MMIP composites [108].

The problem faced in the formation of Fe_3_O_4_ NPs is that nanoscale particles with a large surface-to-volume ratio will cause aggregation during particle formation [106]. In an effort to improve the stability and biocompatibility of Fe_3_O_4_ NPs, surface modification of core-shell NPs was carried out using biopolymers such as chitosan, cyclodextrin, etc. The resulting chitosan-Fe_3_O_4_ composite not only provided support but also acted as a functional monomer during the preparation of the MIP. Chitosan is the most commonly used modifier because of its high natural abundance and because it is biodegradable, biocompatible and non-toxic. The use of chitosan in MMIP synthesis also introduces several functional groups, such as amino and hydroxyl groups, which provide flexibility for the structural modification and help in creating more specific imprinting sites on MMIPs for target analytes [109].

The extraction of tricyclazole from rice and water samples was carried out by Laskar et al. using chitosan-based MMIPs [31], which showed high selectivity and specificity compared to the MNIP. The MMIP showed adsorption equilibrium within 30 min and a maximum binding capacity of 4579.9 µg/g; the Q MMIP was 45,454.55 µg/g and the Q MNIP was 26,315.79 µg/g, with recovery percentages of 89.4% (rice) and 90.9% (water), respectively [31,109]. Yuwei et al. [110] prepared magnetic chitosan NPs by chemical co-precipitation of Fe^2+^ and Fe^3+^ ions using sodium hydroxide (NaOH) in the presence of chitosan, followed by hydrothermal treatment for Cu(II) removal. The maximum absorption capacity (Qm) of Cu(II) was calculated to be 35,500 µg/g.

##### Silica

**Figure 4:**
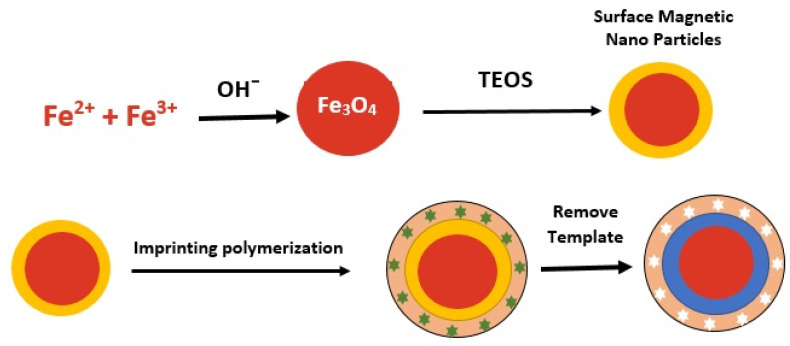
The preparation of MMIPs using silica.

Silica-coated Fe_3_O_4_ (Fe_3_O_4_@SiO_2_) is also a common choice because the SiO_2_ layer is a good medium for immobilizing different functional groups [43]. Coating materials, including polymers, Au and silica have been developed to modify MNPs. Among these materials, silica is one of the most ideal coating media for magnetic materials. The chemical nature of silica is inert, which prevents it from affecting the redox reactions at its core. With a suitable coating, the dipole-dipole magnetic attraction between the NPs can be covered, which can minimize or prevent aggregation [111]. Several studies have been conducted using silica as a coating material to produce stable polymers and good recovery. The preparation of MMIP using silica can be seen in Figure 4.

MMIPs were also used by Chen et al. as a SPE adsorbent in the determination of resveratrol in wine samples [25]. In order to avoid oxidation and provide a biocompatible and hydrophilic surface, the surface of the Fe_3_O_4_ NPs was encapsulated with silica. Surface modifications were carried out with silanol, through a covalent attachment mechanism of specific ligands on the surface of Fe_3_O_4_@SiO NPs from the silanol group. The MMIP showed a recovery of spiked samples ranging from 79.3% to 90.6%, with a detection limit of 4.42 ng/mL.

Karimi et al. synthesised adsorbent silica-coated MNPs to remove humic acid from water sources, resulting in easier and faster separation from solution in the presence of a magnetic field [111]. The maximum monolayer adsorption capacity using the Langmuir isotherm model for MNPs and silica-coated MNPs was 196,070 μg/g and 96,150 μg/g, respectively.

MMIP Fe_3_O_4_@SiO_2_-MIPs were made Dil et al. for dispersive magnetic solid phase micoextraction (d-MSP-μ-E), in order to design an easy and effective method for the extraction of melatonin from a methanol extract of *Portulaca oleracea* [112]. The selectivity of MMIP for melatonin using seven different analogues (tryptophan, serotonin, ferulic acid, mefenamic acid, quercetin, luteolin and chlorogenic acid) indicated that the MMIP had the highest capacity for melatonin among the analogues, with its capacity being in the order melatonin > tryptophan > serotonin > ferulic acid > mefenamic acid > quercetin > luteolin > chlorogenic acid. Hiratsuka et al. [22] showed that MMIP had a higher selective absorption capacity for melatonin compared to the others, with selectivity factor values (β) of 1.60 for tryptophan, 1.68 for serotonin, 2.02 for ferulic acid, 2.38 for mefenamic acid, 2.32 for quercetin, 2.40 for luteolin and 2.50 for chlorogenic acid. A selectivity value of more than 1 indicates that the MMIP has selectivity for melatonin. The Qmax value of the MMIP was 109,100 μg/g, which was higher than the MNIP (39,040 μg/g) [112,113]. This behaviour is based on the point of view that the seven competing analogues do not have a strong impact on entering the mould cavity, possibly due to their size being much smaller or larger than the mould cavity produced by melatonin [112,114,115].

Table 5 shows that modification with oleic acid is more widely used. Based on the study of Gao et al. [34], the surface of the iron atoms coordinates with the carboxylic acid group of the oleic acid ligand, forming a steric stabilising layer that prevents the aggregation of NPs and facilitates the formation of monodispersed samples. The results of surface modification on the organic phase show that the carboxylate and amino groups were able to stabilise the magnetite surface to produce smaller NPs.

#### 3.2.2. Effect of Initial Concentration of FeCl_3_ and the Molar Ratio of Surfactant

In general, smaller and more uniform NPs indicate that the protective reagent interacts more strongly with the NPs, forming a more stable protective layer. Yan et al. [34] investigated the effect of the initial concentration of FeCl_3_, the molar ratio of FeCl_3_ and the shielding agent on the size of the NPs. By modifying the solvothermal procedure using a surfactant mixture of polyethylene glycol (PEG 6000) and sodium dodecyl sulphate (SDS), they succeeded in synthesising smaller and more uniform Fe_3_O_4_ NPs in large quantities, showing that a mixture of SDS and PEG can act as a shielding reagent, shielding more efficiently than PEG alone. Fe_3_O_4_ NPs were obtained by the solvothermal method using PEG and/or SDS as the shielding agents to prevent particles from aggregating after the Fe_3_O_4_ synthesis process. The molar ratio between the total shielding reagent and the FeCl_3_ was established as 11:3 (with the shielding agents consisting of 4 mmol SDS and 7 mmol PEG repeat units), and the nanoparticle size increased as the initial concentration of FeCl_3_ increased.

The initial concentration of FeCl_3_ is a very important factor that determines the particle size. A larger particle size was obtained when an initial molar ratio of 11:3 was used, while a lower initial concentration of FeCl_3_ (0.75 mmol) and a shorter growth time (24 h) resulted in the mean nanoparticle size decreasing to about 15 nm. When the concentration of FeCl_3_ was increased to 6.0 mmol with the same reaction time, Fe_3_O_4_ NPs with a larger size (~190 nm) were obtained [34]. The molar ratio between the shielding reagent and reactant is also a very important factor in determining the particle size. When using the initial concentration of reactants and changing the concentration of the protective reagent, results showed that the NPs became smaller (from 50 to 30 to 20 nm, respectively) as the molar ratio between SDS and FeCl_3_ increased (from 4:3 to 5:3 to 6:3, respectively). It is well known that the NPs are protected more thoroughly, immediately after formation, as the amount of shielding reagent increases, and the particle size should therefore be smaller [34].

### 3.3. Factors in the Synthesis of MMIP Using Polymerisation Components

The third step in the making of MMIP is surface-imprinted polymerisation using NPs that serve as a magnetic core in the presence of the template molecules, functional monomers and cross-linkers. The MMIP synthesis methods that have been carried out are suspension polymerisation, emulsion polymerisation and surface printing polymerisation, among others [117].

The polymer is the most important part in MIP and MMIP and determines the attachment to the template molecule. To synthesise selective MIPs for a single analyte, it is important to determine the template properties, functional monomers, cross-linkers, solvents, polymerisation initiators and even the polymerisation method initiation and duration. In polymerisation, the master molecule is dissolved in a selected solvent called a porogen, together with a functional monomer capable of polymerisation [118]. In fabricating the desired MMIP, the active group (such as a carbon-carbon double bond) will first be grafted onto the MNP for better surface immobilization. Once the initiator is added, the process starts between the surface grafted active groups, the monomers and the cross-linkers [43].

The synthesis of MMIP was carried out by reacting the modified magnetic core-shell with the MIP components. The step begins with pre-polymerisation between the template and the functional monomer [27]. Where the synthesis of Fe_3_O_4_ MNPs has previously been carried out, a modification step (using SiO or oleic acid) is then carried out to increase the stability of the MNPs and protect the particles from aggregation. The modified Fe_3_O_4_ is then added to the polymer solution, the final mixture is cooled, and the obtained Fe_3_O_4_@SiO@MIP is separated by an external magnetic field. The particles are washed several times with acetonitrile and another 5 times with methanol and acetic acid to remove the template. The template removal is monitored by ultraviolet-visible spectrophotometer and high-performance liquid chromatography at 253 nm, and the MMIP is washed with deionized water until the eluent becomes neutral [119]. The interaction between templates and functional monomers is more stable when a strong template-monomer complex is formed, which results in a high printing factor [27].

Functional monomers are important factors for binding interactions in MIT, affecting the affinity of the MIP binding sites, which interact with template molecules on MIP pre-polymerisation [120]. The formation of a stable template-monomer complex is critical for the success of MIPs [37]. The amount of functional monomer used can also affect the binding capacity between the monomer and the template [27]. In a study by Tom et al. [37], the highest IF value (3.92) was achieved with a polymer having a monomer: template ratio of 6:1, with a cross-linker ratio of 20. When using a ratio of 15:1, the excess functional monomer reduced the IF value to 1.14. This indicates very little template-specific retention of the unretained compound compared to the NIP and shows that increasing the number of monomers, with a decrease in the number of cross-linkers in the polymerisation mixture, will increase the imprinting efficiency of the MIP.

The cross-linker also plays an important role with regards to the selectivity of the MIP. The effect of the template: the cross-linker ratio is also related to the effectiveness of the cavity in the polymerisation mixture. The ratio for template and cross-linker of 1:40 is used when setting up a non-covalent MIP, as this provides rigidity to the polymer network, which helps ensure cavities that are complementary in form, as well as ensuring template functionality. The most common cross-linker is ethylene glycol dimethacrylate, with the highest selectivity occurring at around 40–60% vol% cross-linker [37]; a higher volume of ethylene glycol dimethacrylate substantially eliminates the imprinting effect, indicating no specific retention of the template [37].

As a medium for the polymerisation reaction, the solvent has a significant effect on the template-monomer interactions. The solvent must interact and dissolve all the starting materials but should not be too distracting during the polymerisation reaction [121]. A study by Dong et al. [38] investigated the effect of solvent on the adsorption selectivity of MIP with theophylline as the template and methacrylic acid as the functional monomer. They compared three solvents, namely chloroform, tetrahydrofuran and dimethyl sulphoxide (DMSO), and found that DMSO had the highest affinity for theophylline and methacrylic acid, but the lowest IF (1.0533) compared to tetrahydrofuran (IF = 1. 1076) and chloroform (IF = 3.3197). Lamaoui et al. [17] also reported that the choice of solvent used in sonochemistry is very important and can affect the reactivity and yield of the product. They [17] conducted a comparative study of the effect of various solvents on the synthesis of MMIPs based on the use of a high-power ultrasound probe against SMX, using DMSO, dimethylformamide, ethanol, acetonitrile and acetone. The MMIP synthesised with DMSO was chosen for analytical applications to detect SMX, as it presented a high dissipated ultrasonic power; the IF values were: DMSO 1.59 ± 0.01, ethanol 2.07 ± 0.01 and dimethylformamide 1.41 ± 0.01, while acetonitrile and acetone were reported to produce no significant polymer and no polymer, respectively.

At the synthesis stage, it is necessary to computationally select monomers and crosslinkers which will then be applied to the synthesis stage, so that it will reduce the time to carry out the trial error process in the synthesis. The things that greatly affect the MMIP synthesis step are the solvent used, the monomer used, and the comparison of the concentration of the template, solvent, and monomer in determining the association constant (Ka) to get the best Ka value. Therefore, it is necessary to conduct a study to find the suitable functional monomer (FM), the ratio of template (T) to FM, and the type of crosslinker [122].

## 4. Conclusions

In the synthesis of MMIPs, it is necessary to achieve the expected conditions by producing smaller and more uniform NPs, so as to form a more stable protective layer.

The first step in making MMIP is the magnetic core step, which is the most crucial step for successful MMIPs. Fe_3_O_4_ is the most widely used magnetic material; when Fe_3_O_4_ was used, the size of the NPs and the MS increased linearly with high temperature and reaction time. The higher the pH in the synthesis of Fe_3_O_4_, the smaller the particle size. The method of synthesising the magnetic core will result in different particle sizes and will determine their compatibility and suitability for a particular application. The co-precipitation method can produce a high yield of magnetite via an easy procedure, but the resulting particle size is irregular. The solvothermal method results in a more uniform size and distribution of magnetite Fe_3_O_4_ particles but involves higher costs and greater effort due to the very high temperatures required in the heating step.

Much effort should be devoted to exploring future MMIPs by considering the following:

1. New processes for nanomaterials and optimization of the modification procedures in the development of MMIP synthesis;
2. Further exploration of surface modification materials, such as chitosan and cyclodextrine or changes to the carboxylate groups and other amines;
3. Discovering other magnetic metals besides the existing ones and modifying the magnetic properties of metals.

## Figures and Tables

**Table 3:** Résumé of advantages and disadvantages of techniques for the magnetic preparation of MMIP.

Method	Advantages	Disadvantages	Ref.
Co-precipitation	A large number of nanoparticles can be synthesised Water-soluble Biocompatible with iron oxide nanoparticles Easy procedure	Particle size is irregular Control of the particle size distribution is limited Broad distribution of sizes Aggregation of particles	[97,98]
Solvothermal	Increasing the effective collision of metal ions Narrow size distribution Resulting in a more uniform size Better dispersion properties	Higher costs Greater effort due to the very high temperatures involved in the heating step	[98]

**Table 4:** Effect of temperature and reaction time in the manufacture of magnetite Fe_3_O_4_.

Factor	Effect [36,99,100]
Reaction temperature	Higher reaction temperature, larger size of nanoparticle [36,99,100], increased magnetisation saturation (MS) [100], more uniform and regular structure [36].
Reaction time	Higher reaction time, larger size of nanoparticle [36,99,100] and increased magnetisation saturation (MS) [100].

**Table 5:** Summary of MMIP methods using modified material.

Analyte	Modification Component	Q MMIP (μg/g)	Q MNIP (μg/g)	Recovery MMIP (%)	Ref.
Chloramphenicol	Oleic acid	5679	2922	84.3–90.9	[8]
Imidacloprid	Oleic acid	24,032	9.97	94.0–98.0	[82]
Metronidazole	Oleic acid	10,800	4920	90.6–104.2 in toner sample; 84.1–91.4 in powder sample; and 90.3–100.4 in cream	[49]
6-mercaptopurine (6-MP) and thioguanine (TG)	Oleic acid	6-MP: 822.29 TG: 519.15	6-MP: 537.92 TG: 352.24	8.89–103.03 for 6-MP and 85.94–98.27 for TG	[21]
Tricyclazole	Chitosan	45,454.55	26,315.79	89.4 (rice), 90.9 (water)	[31]
Cu(II)	Chitosan	35,500	-	-	
Resveratrol	Tetraethoxysilane (TEOS)	5331.92	-	79.3–90.6	[70]
Humic acid	Tetraethoxysilane (TEOS)	196,070	96,150	-	[25]
Melatonin	Tetraethoxysilane (TEOS)	109,100	39,040	93.07–104.1	[116]

## Data Availability

Data sharing not applicable.

## Abbreviations

3-APTES	Aminopropyltriethoxysilane
DMSO	Dimethyl Sulphoxide
FM	Ferrimagnetic
FRP	Free Radical Polymerisation
IF	Imprinting Factor
LOD	Limit Of Detection (LOD)
LOQ	Limit Of Quantification (LOQ)
MIP	Molecular Imprinted Polymer
MIT	Molecular Imprinted Technology
MMIP	Magnetic Molecularly Imprinted Polymer
MMI-SPE	Magnetic Molecular Imprinted Solid Phase Extraction
MNIP	Magnetic Non-Imprinted Polymer
MNPs	Magnetic Nanoparticles
MPS	3-(Trimethoxysilyl) Propyl Methacrylate
MS	Magnetisation Saturation
MSPE	Magnetic Solid Phase Extraction
MTEOS	Methacryloxypropyltrimethoxysilane
NIP	Non-Imprinted Polymer
NPs	Nanoparticles
PEG	Polyethylene Glycol
Q	Maximum Adsorption
SDS	Sodium Dodecyl Sulphate
SPE	Solid Phase Extraction
SPM	Superparamagnetic
TEM	Transmission Electron Microscopy
TEOS	Tetraethyl Orthosilicate
TPA	Terephthalic acid
VSM	Vibrating Sample Magnetometer
XRD	X-ray Diffraction
